# Sperm Preservation by Freeze-Drying for the Conservation of Wild Animals

**DOI:** 10.1371/journal.pone.0113381

**Published:** 2014-11-19

**Authors:** Takehito Kaneko, Hideyuki Ito, Hidefusa Sakamoto, Manabu Onuma, Miho Inoue-Murayama

**Affiliations:** 1 Institute of Laboratory Animals, Graduate School of Medicine, Kyoto University, Kyoto, Japan; 2 Kyoto City Zoo, Kyoto, Japan; 3 Wildlife Research Center of Kyoto University, Kyoto, Japan; 4 Center for Environmental Biology and Ecosystem studies, National Institute for Environmental Studies, Ibaraki, Japan; University Hospital of Münster, Germany

## Abstract

Sperm preservation is a useful technique for the maintenance of biological resources in experimental and domestic animals, and in wild animals. A new preservation method has been developed that enables sperm to be stored for a long time in a refrigerator at 4°C. Sperm are freeze-dried in a solution containing 10 mM Tris and 1 mM EDTA. Using this method, liquid nitrogen is not required for the storage and transportation of sperm. We demonstrate that chimpanzee, giraffe, jaguar, weasel and the long-haired rat sperm remain viable after freeze-drying. In all species, pronuclei were formed after the injection of freeze-dried sperm into the mouse oocytes. Although preliminary, these results may be useful for the future establishment of “freeze-drying zoo” to conserve wild animals.

## Introduction

Many animal species are endangered or threatened with extinction [1]. However, assisted reproductive techniques can help species that are experiencing reproductive difficulties in captive and wild conditions [2]. Gamete preservation, especially sperm, is one of the effective methods in assisted reproductive techniques. Although, cryopreservation has been used as a standard method for storing sperm, there are a lot of animals for which a freezing and collecting method for sperm has not been established. Furthermore, the continuous supply of liquid nitrogen and mechanical maintenance of equipment is required for storing frozen sperm. Valuable sperm samples that are stored in liquid nitrogen might be lost if liquid nitrogen supplies cease, especially during disasters such as earthquakes and typhoons. Therefore, safe facilities and equipment are needed to store frozen genetic resources and prevent unexpected accidents [3].

Freeze-drying is the ultimate method for storing biological material. Using this method, samples can be kept for a long time in a refrigerator (4°C) or at ambient temperature [4], [5]. Freeze-dried sperm can also remain viable, and successful results have been reported for various mammals [6]–[11]. In the mouse and rat especially, freeze-drying methods have improved, resulting in high fertility [12]–[16]. At present, freeze-drying methods enable mouse and rat sperm to be preserved for 3 to 5 years at 4°C in a solution containing 10 mM Tris and 1 mM ethylenediaminetetraacetic acid (EDTA) (TE buffer) [4], [5]. Furthermore, freeze-dried sperm can be easily and safely transported worldwide at ambient temperature [17]. In addition, short term preservation at ambient temperature that requires neither liquid nitrogen nor dry ice is possible [18]. In this study, we freeze-dry sperm collected from chimpanzee, giraffe, jaguar, weasel and the long-haired rat, and then estimate the viability of the sperm to apply freeze-drying of sperm for conserving wild animals.

## Materials and Methods

### Media

TE buffer (10 mM Tris, 1 mM EDTA, pH 8.0; Applied Biosystems/Ambion, Austin, TX, USA) was used as a solution for freeze-drying the sperm [4], [5], [14]. The medium used for manipulation, including the collection and handling of oocytes and ICSI, was H-CZB medium, a modified CZB medium [19], [20] with 20 mM Hepes-Na, 5 mM NaHCO_3_ and 0.1 mg/ml polyvinyl alcohol (cold water soluble; M_r_ 30,000–70,000) added instead of bovine serum albumin [21].

### Sperm collection

A chimpanzee (*Pan troglodytes*, 20 years old), reticulated giraffe (*Giraffa camelopardalis reticulata*, 13 years old), and jaguar (*Panthera onca*, 25 years old) kept in the Kyoto City Zoo were used in this study. Animal care and procedures of chimpanzee referred to the Guidelines for Association of Zoos and Aquariums (AZA). Chimpanzees were maintained in the room (36.94–43.87 m^2^) with feeding (3 times/day). Environmental enrichments such as a tower (9 m), trees and artificial ant-hill were prepared outside. Samples from jaguar came from a specimen that died by circulatory failure. Samples from the Japanese weasel (*Mustela itatsi*, age-indeterminate) came from a specimen that died at the wildlife Rescue Center of Kyoto City Zoo after rescue from traffic accident. Sperm was also obtained from a road-killed Ryukyu long-haired rat (*Diplothrix legata*, age-indeterminate) kept in the National Institute for Environmental Studies. It was found on the road at Kunigami, Okinawa, Japan. In this study, animals that are specified for threatened species and can be collected sperm in zoo or field were used. In the chimpanzee and giraffe, sperm ejaculated naturally on the floor were collected in a plastic bag. Testis and epididymides of the jaguar and weasel were collected after the death of an individual at the Kyoto City Zoo. These sperm and tissues were transported to Kyoto University within 10 min. Testis and epididymides of the long-haired rat were also transported at 4°C to Kyoto University by delivery service.

### Freeze-drying sperm

**Figure 1:**
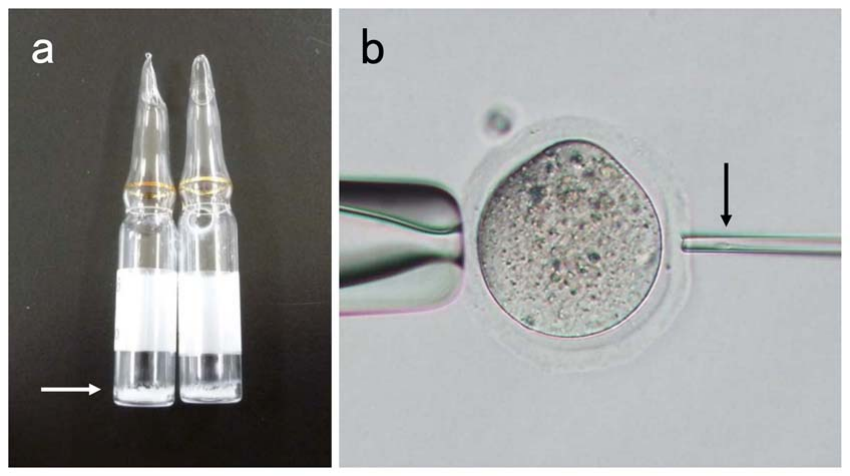
(a) Glass ampoules with freeze-dried giraffe sperm. Arrow shows the dried sperm on the bottom of the ampoules. (b) Sperm injection into mouse oocytes. Arrow shows giraffe sperm drawn into the injection pipette.

Freeze-drying of sperm was carried out using the same method for mouse and rat described by Kaneko [4], [5], [17]. In the chimpanzee and giraffe, each ejaculated sperm sample was diluted with TE buffer, and transferred to a 1.5 ml microcentrifuge tube for centrifugation at 800×G for 10 min. The supernatant was removed, and the sperm mass was re-suspended in 1 ml of fresh TE buffer. Jaguar, weasel and the long-haired rat sperm collected from cauda epididymides were suspended in 1 ml of TE buffer in a 1.5 ml microcentrifuge tube. Aliquots of 100 µl of sperm suspension were transferred into long-necked glass ampoules for freeze-drying (651506, Wheaton, Millville, NJ, USA). Ten ampoules of each animal were plunged into liquid nitrogen for 20 s and then connected to the manifold of a freeze-drying machine (Freeze-drying systems 77530, Labconco, Kansas City, MO, USA). The sperm suspension was dried for 4 h at a pressure of 0.04 hpa. All ampoules were flame-sealed and stored at 4°C (Fig. 1a). Freeze-dried sperm were stored for 1 month.

### Oocyte collection

B6D2F1/Crlj female mice 8–16 weeks old (Charles River Japan Inc., Yokohama, Japan) were used as oocyte donors. Animals were housed in plastic cages in a specific pathogen-free barrier facility that was air-conditioned (temperature 24±1°C, humidity 40±10%) and light-controlled (lights on from 08:00 to 20:00). All animal care and procedures performed in this study conformed to the Guidelines for Animal Experiments of Kyoto University, and were approved by the Animal Research Committee of Kyoto University.

Females were induced to superovulate by an intraperitoneal injection of 5 IU of pregnant mare serum gonadotropin (ASKA Pharmaceutical. Co., Ltd., Tokyo, Japan) followed by an injection of 5 IU of human chorionic gonadotropin (ASKA Pharmaceutical. Co., Ltd.) 48 h later. Cumulus-oocyte complexes were collected from oviducts 13 to 15 h after the injection of human chorionic gonadotropin, and oocytes were freed from cumulus cells by treatment with 0.1% hyaluronidase in H-CZB medium for 5 min. Oocytes were rinsed in fresh H-CZB medium and kept at room temperature before sperm injection.

### Intracytoplasmic sperm injection (ICSI)

**Figure 2:**
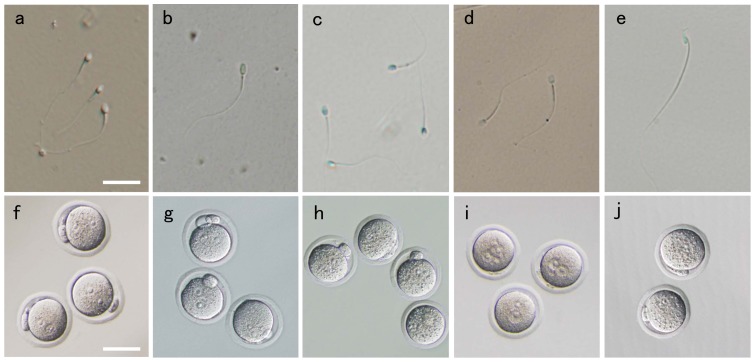
Freeze-dried sperm of (a) chimpanzee, (b) giraffe, (c) jaguar, (d) weasel and (e) the long-haired rat after rehydration. The common scale bar for a–e = 20 µm . Oocytes formed pronuclei after injection of the freeze-dried sperm of (f) chimpanzee, (g) giraffe, (h) jaguar, (i) weasel and (j) the long-haired rat. The common scale bar for f–j = 50 µm.

ICSI was carried out using the method previously described by Kaneko [4], [5], [17] (Fig. 1b). One freeze-dried ampoule of each animal was used, and rehydrated sperm by adding 100 µl of sterile distilled water (Fig. 2a–e). A small volume (1–2 µl) of the sperm suspension was mixed thoroughly with a droplet of H-CZB medium containing 12% (w/v) polyvinylpyrrolidone (M_r_ 360,000, ICN Pharmaceuticals, Costa Mesa, CA, USA). Sperm of normal shape were selected and placed into another droplet of H-CZB medium containing 12% polyvinylpyrrolidone. A single spermatozoon was drawn by its tail into the injection pipette. Oocytes were placed in a droplet of H-CZB medium. The zona pellucida of oocytes held with a holding pipette was opened by applying a few piezo pulses. The oolemma was then opened with piezo pulses, and a sperm was introduced into the oocyte. Sham injection was carried out for control. Microinjected oocytes were cultured for 6–8 h in H-CZB medium at 37°C under 5% CO_2_ and 95% air. Oocytes containing two distinct pronuclei were recorded as being fertilized.

## Results

In all animal used in this study, collected sperm were already immotile. However, these showed morphologically normal shape before freeze-drying, and most of freeze-dried sperm did not separate tail, and maintained normal shape after rehydration (Fig. 2a–e). Table 1 shows the number and percentage of oocytes forming pronuclei after injection with freeze-dried chimpanzee, giraffe, jaguar, weasel and the long-haired rat sperm. No pronuclei appeared in the oocytes after sham injection. Of 29 oocytes injected with chimpanzee sperm, 14 (48%) oocytes survived, and 12 (86%) oocytes formed two distinct pronuclei (Fig. 2f). In the giraffe, all surviving oocytes (12 = 50% of 24) injected with sperm formed two pronuclei (Fig. 2g). Of 34 oocytes injected with jaguar sperm, 23 (68%) oocytes survived, and 22 (96%) formed two distinct pronuclei (Fig. 2h). All surviving (12 = 43% of 28) oocytes injected with weasel sperm formed two pronuclei (Fig. 2i). Of 19 oocytes injected with long-haired rat sperm, 11 (58%) survived and 10 (91%) formed two distinct pronuclei (Fig. 2j).

**Table 1:** Pronuclear formation of oocytes injected with freeze-dried sperm of various animals.

Animals	No. of oocytes injected	No. (%) of surviving oocytes ^a^	No. (%) of oocytes forming pronuclei ^b^
Sham injection	30	24 (80)	0 (0)
Chimpanzee	29	14 (48)	12 (86)
Giraffe	24	12 (50)	12 (100)
Jaguar	34	23 (68)	22 (96)
Weasel	28	12 (43)	12 (100)
Long-haired rat	19	11 (58)	10 (91)

The percentages were calculated from no. of ^a^surviving oocytes/oocytes injected, and ^b^oocytes forming pronuclei/surviving oocytes.

## Discussion

We freeze-dried sperm collected from chimpanzee, giraffe, jaguar, weasel and the long-haired rat, and demonstrated that pronuclei were formed when these sperm were injected into the mouse oocytes. This is the first report that the viability of sperm collected from wild animals can be maintained after freeze-drying. In this study, pronuclei were formed in the mouse oocytes injected with freeze-dried sperm of different animal species (Table 1, Fig. 2f–j). Mouse oocytes have been used previously for fertility tests of sperm collected from various mammals [22], [23]. Pronuclei appeared in mouse oocytes when injected with sperm of various mammals by ICSI. It is well known that removal of the zona pellicida of hamster oocytes allows penetration of sperm of other mammals and pronuclei then form after penetration of sperm [24]. The results of this study further demonstrated that mouse oocytes could receive the sperm of various animals by ICSI, even wild animals such as chimpanzees, giraffes, jaguars, weasels and the long-haired rats. Although the oocytes collected from same animal species are required for detail analysis of freeze-dried sperm, mouse oocytes can be used as simple tool to estimate sperm viability in various species in which a method for collection of oocytes has not been established.

In mouse and rat, the viability of freeze-dried sperm is protected by using TE buffer, allowing sperm to be stored for a long time after freeze-drying [4], [5]. Successful freeze-drying of rabbit and hamster sperm using a solution of similar composition to TE buffer has also been reported [8], [10]. The epididymal sperm is tolerant of physical stress, as DNA is tightly condensed with -SS- bonds [25]. Furthermore, pH and a small volume of EDTA in the solution are helpful to protect sperm from physical stress during freeze-drying [12], [14]. Although further detail studies are required, it was thought that TE buffer also protected chimpanzee, giraffe, jaguar, weasel and the long-haired rat sperm from damage during freeze-drying.

In this study, we collected chimpanzee and giraffe sperm that had been ejaculated naturally onto the floor. These sperm were already immotile before freeze-drying. Sperm also became immotile after freeze-drying even if fresh motile sperm were used. ICSI is an available technique that can fertilize oocytes with immotile sperm that have been freeze-dried or collected from dead animals [26]. The combination of assisted reproductive techniques by freeze-drying sperm and ICSI is a powerful tool for increasing animal populations [27]. Further study using sperm collected from various animals lead to efficient freeze-drying method using TE buffer as a useful tool for sperm preservation of endangered species.

Ideally, sperm and oocytes from a species should be collected for assisted reproduction of endangered species. However, the timing of oocyte collection is seasonally limited, and there are considerable difficulties in the collection of sperm and oocytes at the same time for *in vitro* fertilization. However, sperm can be preserved temporarily because sperm collection is easier than oocyte collection. Oocytes can be fertilized immediately after collection by using preserved sperm. Sperm preservation is useful for applying assisted reproduction to endangered species. Freeze-drying, especially, is relatively easy and sperm can be transported safely without using liquid nitrogen and dry ice. We believe that a “freeze-dry zoo” is the ultimate method to protect wild species from extinction, not only mammals but also birds, reptiles, amphibians, and fishes.
